# Exosome in Crosstalk between Inflammation and Angiogenesis: A Potential Therapeutic Strategy for Stroke

**DOI:** 10.1155/2022/7006281

**Published:** 2022-08-23

**Authors:** Yongdan Cun, Yaju Jin, Danli Wu, Li Zhou, Chengcai Zhang, Simei Zhang, Xicheng Yang, Zuhong Wang, Pengyue Zhang

**Affiliations:** ^1^Key Laboratory of Acupuncture and Massage for Treatment of Encephalopathy, College of Acupuncture, Tuina and Rehabilitation, Yunnan University of Traditional Chinese Medicine, Kunming 650500, China; ^2^Acupuncture Department, Kunming Traditional Chinese Medicine Hospital, Kunming 650500, China

## Abstract

The endothelial dysfunction, associated with inflammation and vascular permeability, remains the key event in the pathogenesis of cerebral ischemic stroke. Angiogenesis is essential for neuroprotection and neural repair following stroke. The neuroinflammatory reaction plays a vital role in stroke, and inhibition of inflammation contributes to establishing an appropriate external environment for angiogenesis. Exosomes are the heterogeneous population of extracellular vesicles which play critical roles in intercellular communication through transmitting various proteins and nucleic acids to nearby and distant recipient cells by body fluids and circulation. Recent reports have shown that exosomal therapy is a valuable and potential treatment strategy for stroke. In this review, we discussed the exosomes in complex interaction mechanisms of angiogenesis and inflammation following stroke as well as the challenges of exosomal studies such as secretion, uptake, modification, and application.

## 1. Introduction

Cerebral ischemic stroke is a common and serious cerebrovascular disease, accounting for 87% of all strokes [1], and is the leading cause of persistent disability. Although the incidence, prevalence, and mortality of stroke tend to decline, the results from the Global Burden of Stroke 2017 study indicated that the overall stroke burden keeps growing worldwide [2]. Ischemic strokes are caused by sudden cerebral vascular obstruction, and cerebral tissue ischemia and hypoxia can lead to mitochondrial dysfunction, decreased adenosine triphosphate (ATP), and excessive production of reactive oxygen species (ROS) after a few minutes [3]. This event results in the dysfunction of the sodium pump, calcium pump, and other ATP-dependent ion transporters, which causes Ca^2+^ excretion obstacles and calcium overload [4]. Calcium overload promotes the release of glutamate, and glutamate increased in the intercellular space causes excessive excitation of glutamate receptors on the postsynaptic membrane, which further increases the loading of intracellular Ca^2+^ and leads to cell necrosis [4]. Besides, after mitochondria were stimulated by calcium overload, the mitochondrial permeability transition pore (MPTP) was opened, and then, cytochrome C and other proapoptotic proteins are released through MPTP, which can lead to cell apoptosis [5]. In addition, when the blood supply to brain tissue is interrupted, ATP synthesis is decreased, and the increase of adenosine monophosphate (AMP)/ATP ratio can directly activate AMP-activated protein kinase and then inhibit the action of the mammalian target of rapamycin (mTOR), leading to cell autophagy [6]. Overall, these complex pathological cascades, such as calcium overload, glutamic acid excitatory toxicity [7], and oxidative stress, will eventually lead to edema, inflammatory reaction, necrosis, autophagy, apoptosis, and other serious consequences for the central nervous system cells [8], which will seriously affect the health and living quality of patients and increase the burden on families and society.

At present, the intravenous recombinant tissue plasminogen activator (IV-rtPA) was considered to be effective for acute stroke [9]; however, it is a pity that the therapeutic time window is only 4.5 hours. The risk might outweigh the benefit when the IV-rtPA was applied beyond 4.5 hours after stroke [10], and the hemorrhagic transformation and oxidative stress are major clinical risks, which will further amplify the pathological cascade [9, 11]. Fortunately, the ischemic penumbra becomes the salvageable target for counteracting the expansion of the infarct core and promoting the recovery of stroke [12, 13].

The interaction between inflammation and angiogenesis exists in many pathological processes, such as tumors, diabetic retinopathy, and stroke. Various proinflammatory cytokines, such as tumor necrosis factor *α* (TNF-*α*), interleukin 4 (IL-4), and monocyte chemoattractant protein 1(MCP-1) can promote the expression of vascular endothelial growth factor (VEGF) and the proliferation of endothelial cells and promote angiogenesis [14]. Similarly, the classical angiogenic factor VEGF also promotes the inflammatory response [15]. The crosstalk among different systems plays a crucial role in maintaining the homeostasis of the central nervous system and the integrity of the blood-brain barrier (BBB) both under physiological conditions and various diseases such as cerebral ischemia [16–19].

Exosomes, with a diameter of about 30-150 nm, are a heterogeneous population of extracellular vesicles equipped with functional molecule-decorated phospholipid bilayer and rich in proteins, lipids, and nucleic acids [20, 21]. As an alternative therapeutic strategy to whole-cell implantation, exosomes play critical roles in intercellular communication through transmitting various proteins and nucleic acids to nearby and distant recipient cells by body fluids and circulation [22]. Recently, exosome-based treatment has shown immense effects in angiogenesis, anti-inflammation, neurogenesis, and antiapoptosis of stroke [23–25].

In this review, we summarize the biogenesis, uptake, and function of exosomes and the mechanisms of exosomes in crosstalk between angiogenesis and inflammation following stroke. And then, we discuss the challenges in the translation of exosome-based therapy to clinical applications.

## 2. The Features of Exosomes Applicable to the Treatment of Stroke

### 2.1. The Advantages of Exosomes for Stroke

There are many treatments for stroke in the clinic, for instant, anticoagulants, blood pressure-lowering, and cholesterol-lowering drugs can prevent stroke; IV-rtPA and endovascular thrombectomy in the acute stage of stroke can timely achieve the blood flow recanalization [26]; some exercise training, physical factor therapies, and other rehabilitation treatments also can promote the restoration of nerve function in the stage of stroke convalescence [27]. However, numerous patients had poor control of the risk factors of stroke or can not arrive at the hospital within the time window of IV-rtPA and thrombectomy, resulting in severe neurological dysfunction. In summary, the available treatment strategies for stroke are limited; therefore, more research must be done on stroke.

Neurorestorative therapies, as adjunctive therapy to improve stroke outcomes, mainly act on nerve cells, endothelial cells, and immune cells to promote neurovascular remodeling and reduce local and systemic inflammation [28]. Cell-based therapies are a neuro repair therapy for stroke by stimulating endogenous neuroplasticity and brain remodeling to promote neurological recovery following stroke [29]. Increasing evidence has indicated that the positive effects of cell-based therapies are mediated by exosomes, which are derived from the administered cells [22]. In addition, the exosomes have been shown that have many advantages [22], for instance, overcome the obstacles of BBB, can not block blood vessels, and can not induce malignant transformation. Therefore, exosomes provide a novel and potential therapeutic strategy for stroke.

### 2.2. Secretion and Regulation of Exosomes

Almost all living cells can secrete exosomes in diverse pathophysiologic environments [30]. Several kinds of central nervous system cells such as microglia, oligodendrocytes, astrocytes, and neural stem cells (NSCs) can secrete exosomes and regulate the occurrence and development of neurological diseases. These central nervous system cell-derived exosomes play a key role in promoting angiogenesis, regulating inflammation, and remodeling the brain following stroke [25, 31]. In addition, mesenchymal stem cells (MSCs) have attracted wide attention in stroke treatment because they can differentiate into neurons, but the MSCs were ≥15 *μ*m in diameter [32], which makes it difficult to target them in the ischemic areas. Fortunately, exosomes have physiological functions similar to their donor cells; therefore, exosomes originated from central nervous system cells and MSCs are considered a potential therapeutic strategy.

Secretion and release of exosomes is a complex process, and multiple signal transduction factors, such as Rab GTPases, endosomal-sorting complex required for transport (ESCRT), and the ESCRT-associated protein ALIX are involved in the regulation of the secretion of exosomes [33–35]. The first step of the formation of exosomes is the cell membrane invagination which forms the early endosomes, and the further invagination of early endosomes wraps the proteins, lipids, and nucleic acids in the cytoplasm and form intraluminal vesicles (ILVs). The ESCRT family participates in the regulation of the molecular composition of the exosomes and the formation of ILVs, such as ESCRT-0, ESCRT-I, and ESCRT-II in charge of the cargoes sorting, and ESCRT-III undertakes the deformation and fission of membrane [36]. In addition, ALIX recruits ESCRT-III to endosomes and promotes sorting. Although the ALIX is an active form of the ESCRT-associated protein, the ALIX/ESCRT-III pathway needs lysophosphatidic acid rather than being dependent on the ESCRT [36].

And then, with the gradual accumulation of ILVs, the early endosomes mature into late endosomes/MVEs, some MVEs fuse with the cell membrane and secrete exosomes through exocytosis, and the other MVEs are degraded by lysosomes [37–39]. The Rab GTPases are a critical family in this process. The conversion of Rab5 to Rab7 regulates the transition from early to late endosomes [36, 40]. Rab27a and Rab27b are specialized in docking MVEs at the plasma membrane [41]. The Rab7 regulates the fusion of MVEs with lysosomes to degrade the ILVs [36]. The Rab31 inhibits MVEs degradation by recruiting TBC1D2B to Rab7 and further suppressing the fusion of MVEs with lysosomes [42]. Currently, the initiation mechanisms of membrane invagination, the contents sorting (random or specific), and the balance between the degradation of MVEs by lysosomes and the formation of exosomes still needed further exploration.

### 2.3. The Uptake of Exosomes in the Central Nervous System

**Figure 1:**
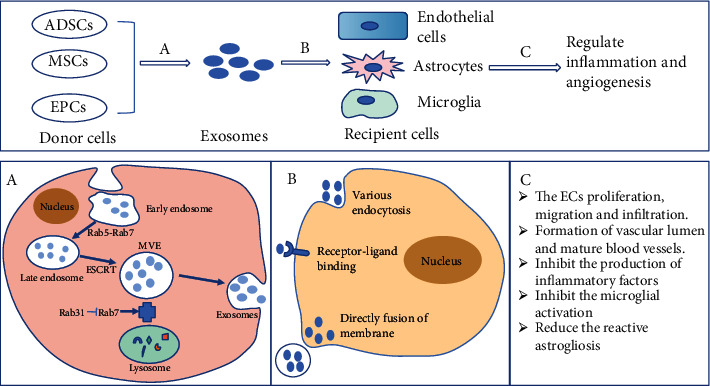
Summary of the exosomal secretion, uptake, and the effect of regulating inflammation and angiogenesis. Process A represents the exosomes derived from donor cells such as ADSCs, MSCs, and EPCs. This complex process is relevant to the formation of early endosomes, early endosomes mature into late endosomes, and MVEs fuse with the cell membrane and secrete exosomes. Process B is exosomes that bind to the central nervous system cells such as microglia, astrocytes, and endothelial cells through specific molecular interactions, the direct fusion of membrane, and various endocytosis. Process C shows the effect of exosomes in regulating inflammation and angiogenesis by promoting the formation of the blood vessel and suppressing the production of neuroinflammatory mediators following stroke. Abbreviations: ADSCs: adipose-derived stem cells; MSCs: mesenchymal stem cells; EPCs: endothelial progenitor cells; MVEs: multivesicular endosomes; ESCRT: endosomal-sorting complex required for transport.

The recipient cells capture the exosomes in many ways, including specific molecular interactions, direct fusion of membrane, and various endocytosis involving macropinocytosis, lipid raft-mediated endocytosis, clathrin-mediated endocytosis, and clathrin-independent endocytosis [43–46]. Recipient cells uptake exosomes by specific molecular such as proteins, sugars, or lipids on the surface of the membrane [47]; for instance, the integrin lymphocyte function-associated antigen 1 on the macrophage-derived exosomes can interact with intercellular adhesion molecule 1 (ICAM-1) on the brain microvascular endothelial cells (BMECs) [48]. The exosomal fusion directly with the membrane of the recipient cells accompanied several key events such as the rearrangement of exosomal membrane proteins, the hydrophobic sequences insert into the target cell membrane, lipid reorganization, protein reconstruction, and membrane dimpling [49]. Macropinocytosis internalizes large amounts of extracellular fluid by forming folds in the plasma membrane, which depends on the functions of actin, Rac1, and Na+/H+ exchanger [43]. The microglia uptake oligodendroglia-derived exosomes by macropinocytosis, and inhibiting the functions of actin, Rac1, and Na+/H+ exchanger resulted in a significant reduction of the uptake of exosomes by microglia [50]. In the process of BMECs uptake of macrophage-derived exosomes, Yuan et al. observed the phenomenon of clathrin-mediated endocytosis and caveolae-mediated endocytosis [48]. In addition, exosomes can also be taken in neurons such as dopaminergic neurons and affect these neurons. After the dopaminergic neurons ingest exosomes, the substantia nigra loss and apoptosis were reduced and the level of dopamine in the striatum was upregulated [51] and restored the homeostasis of oxidative stress, neuroinflammation, and cell apoptosis in the Parkinson's disease model mice [52]. Interestingly, exosomes secreted from stimulated by glutamatergic synapses cortical neurons and were specifically endocytosed by neurons [53]. The formation of exosomes, uptake by central nervous cells, and their effects are shown in Figure 1.

In general, several mechanisms of exosomal uptake have been identified, but the specificity of exosomes to recipient cells and the membrane molecules that participate in the recognition of exosomes by recipient cells still needed further investigation.

## 3. Exosomes in Inflammation following Stroke

### 3.1. Inflammatory Response following Stroke

Following cerebral ischemia, danger-associated molecular patterns, including high-mobility-group box 1 (HMGB1), peroxiredoxins, IL-33, mitochondrial transcription factor A, and cytochrome C, are released by damaged neurons in the minutes and bind to receptors on immune cells and then result in inflammation and neurotoxicity [54–56]. Activated microglia produce distinct phenotypes, M1 type secretes a plentiful of proinflammatory factors including IL-1*β*, IL-23, and TNF-*α*, which aggravate the inflammation [57]. Contrary to the M1 type, the M2 type plays an anti-inflammation role in ischemic injury against oxygen and glucose deprivation [58]. Moreover, circulating immune cells including neutrophils, monocytes/macrophages, and T cells also aggravate central nervous system cell death due to the permeability of BBB increased after cerebral ischemia [54]. At the same time, cellular adhesion molecules on leukocytes and cerebral endothelial cells and cytokines were upregulated following stroke and then promote adhesion and leukocyte transendothelial metastasis, which further aggravates inflammatory response [54, 59]. The astrocytes also produce two distinct phenotypes which, respectively, are A1 and A2 under different pathological conditions. Research has shown that the A1 type was induced by activated microglia under neuroinflammation, while the A2 type was induced by ischemia and plays a neuroprotective role via upregulating neurotrophic factors to promote neuronal survival and function repair [60, 61]. But the remarkable thing is that neuroinflammation can contribute to the enlargement of infarcts; meanwhile, it can promote remodeling and repair at certain stages following stroke [54, 62].

### 3.2. Exosomes in the Inflammatory Response following Stroke

Exosomes participate in the remodeling process by delivering therapeutic nucleic acids, proteins, and drugs affecting inflammation following stroke. The MSC-derived exosomes packaged miR-542-3p target the Toll-like receptor 4 (TLR4) in HA1800 cells (human glial cells) and then prevent ischemia-induced glial cell inflammatory response [63]. Meanwhile, in the inflammation following stroke-induced reactive astrogliosis, the MSC-derived exosomes target astrocytes and reduce the reactive astrogliosis and inflammation; the undergoing mechanism might be related to nuclear factor erythroid-derived 2 (Nrf2)/nuclear factor-*κ*B (NF-*κ*B) [64]. Bone MSC- (BMSC-) derived exosomes with miR-138-5p target the lipocalin 2 (LCN2) on astrocytes and reduce the neurological impairment by inhibiting the astrocytes' inflammatory response and apoptosis after stroke [65]. Melatonin is an effective free radical scavenger and antioxidant, and melatonin-treated plasma exosomes significantly protect against ischemia-induced inflammatory response and inflammasome-mediated pyroptosis via regulating the TLR4/NF-*κ*B pathway [66]. The neural progenitor cell- (NPC-) derived EVs showed the strong suppression of the inflammatory response following cerebral ischemia, and the effect is related to a set of miRNAs packaged in the EVs that inhibited the inflammation-related pathway mitogen-activated protein kinase (MAPK) [67].

### 3.3. Function of Exosomal miRNAs in Inflammation Response following Stroke

Except for exosomal miRNAs, there are many proteins and DNA also involved in some crucial biological processes. For instance, tetraspanin (CD9, CD63, and CD81) and integrin proteins are essential for cell targeting and adhesion, Rab GTPases, annexins, and flotillin contribute to membrane fusion, and G proteins and kinases are signal transduction molecules [20, 68]. Furthermore, in a few types of research, genomic and mitochondrial DNA has been found in exosomes [69]. However, there are few studies on exosomal proteins and DNA in the field of neuroinflammation and angiogenesis following stroke, and increasing evidence has suggested that the exosomal miRNAs are largely responsible for the therapeutic effects [70, 71].

Recent research suggests that exosomal miRNAs have dual effects [71]. On the one hand, the exosomal miRNAs negatively regulate the expression of target genes; for instance, M2 microglia-derived exosomes attenuate ischemic brain injury and promote neuronal survival via exosomal miR-124 which negatively regulates its target gene ubiquitin-specific protease 14 [72]. On the other hand, the exosomal miRNAs as a ligand binds to the receptor; for instance, the exosomal miR-21 and miR-29a have demonstrated that bind to TLR and induce TLR8-mediated activation of NF-*κ*B and NF-*κ*B-mediated secretion of proinflammatory cytokines TNF-*α* and IL-6 [73].

In addition to exogenous miRNAs, exosomal miRNAs are also critical in regulating inflammation following stroke. Microglia are prominent resident immune cells in the central nervous system, and the activated microglia trigger severe inflammatory responses following stroke. The MSC-derived exosomal miR-223-3p inhibits M1 microglia polarization and inflammatory response [74]. Moreover, the human umbilical cord MSC- (hUMSC-) derived exosomes also attenuate microglia-mediated neuroinflammation and promote the recovery of neural function, and the undergoing mechanism is involved in that exosomal miR-146a-5p regulates the IL-1 receptor-associated kinases 1 (IRAK1)/TNF receptor-associated factor 6 (TRAF6) [75]. Serum exosomes from acute cerebral infarction patients aggravate cerebral inflammation and promote microglia activation in middle cerebral artery occlusion (MCAO) rats, and the undergoing mechanism might be related to the exosomal miR-27-3p which targets the peroxisome proliferator-activated receptors *γ* (PPAR*γ*) [76]. The PPAR*γ* is a ligand-activated transcriptional factor that participates in regulating a variety of signaling networks including inflammation, glucose homeostasis, and cell fates [77]. BMSC-derived exosomal miR-221-3p can target the activated transcription factor 3 (ATF3) and then attenuate the neuroinflammation and apoptosis following stroke [78].

While plentiful studies have proved that exosomes can regulate inflammation after stroke, current studies have mainly focused on the miRNAs of exosomes. The effect of other exosomal cargoes on inflammation after stroke needs further study.

## 4. Exosomes in Crosstalk between Inflammation and Angiogenesis following Stroke

### 4.1. Crosstalk between Inflammation and Angiogenesis following Stroke

Angiogenesis usually occurs in the inflammatory environment, the inflammatory response is usually accompanied by the initiation, progression, and stability of angiogenesis [79]. Increasing research has indicated that many immune cells and cytokines not only secrete inflammatory factors to aggravate brain injury but also promote angiogenesis and BBB repair in a later stage [80, 81]. It is well-known that the M2 type microglia affect angiogenesis and anti-inflammation through secreting the IL-10, transforming growth factor *β* (TGF-*β*), insulin-like growth factor (IGF), and VEGF [57]. In the early phase of stroke, the monocytes destroy BBB integrity and exacerbate neuroinflammation through secreting ROS, cytokines, and chemokines [80]. However, the function and phenotype of monocytes are not unchangeable; following differentiation into mature macrophage cells, the monocyte-derived M2 type macrophage cells have a stronger ability in angiogenesis by upregulating the basic fibroblast growth factor (FGF), IGF-1, and placental growth factor (PGF) [80, 82]. The pericytes were considered immune cells in the brain, and the pericytes' membrane protein TLR4 can bind the HMGB1 and induce the secretion of proinflammatory cytokines and chemokines shortly after stroke. Moreover, pericytes are essential for the whole process of angiogenesis including degrading the basement membrane surrounding endothelial cells, promoting the formation of tip cells by endothelial cells, and preventing the degradation of matrix proteins of neovascularization by secreting matrix metalloproteinase, VEGF, and tissue inhibitor of metalloproteinase-3 [83–85].

In addition, the proinflammatory cytokines such as TNF-*α*, MCP-1, and SDF-1 also can affect angiogenesis. The research has shown that the interaction of TNF-*α* with TNF receptor 1 can amplify the effect of erythropoietin-induced angiogenesis by upregulation of the erythropoietin receptor [86]. Significantly, a high dose of TNF inhibits angiogenesis, but the pericytes can enhance angiogenesis and overcome the inhibition of angiogenesis induced by a high dose of TNF [79]. The MCP-1 is a key regulatory molecule of monocytes trafficking to sites of inflammation; meanwhile, the MCP-1 is a chemokine with angiogenic properties. The angiogenic effect of MCP-1 is maintained and modulated by VEGF [87]. The stromal cell-derived factor-1 (SDF-1), also called C-X-C motif chemokine ligand 12 (CXCL12) binds to the CXC receptor 4 (CXCR4) as an inflammatory initiator in the acute phase of stroke [88]. During the postacute phase of stroke, the CXCL12 promotes angiogenesis by recruiting circulating endothelial progenitor cells (EPCs) [89]. In addition, SDF-1 promotes neurogenesis and angiogenesis through CXCR4-mediated downstream protein kinase B (AKT), extracellular signal-regulated kinases (ERK), and P38 MAPK signaling pathways [88, 90].

Similarly, angiogenic factors and growth factors can induce inflammatory responses [81]. Endothelial cell-specific molecule 1 (Esm1) has dual effects on angiogenesis and inflammation, and the research has shown that knockout Esm1 can reduce vascular permeability and cerebral edema following stroke [91]. Moreover, gathered VEGF directly participates in the proliferation, migration, and differentiation of endothelial cells [92, 93], but VEGF is also a chemokine that causes an increase in vascular permeability and inflammation. Clinical evidence has shown that circulating VEGF was elevated 24-48 h after acute stroke and conveyed severe prognostic information [94]. HIF-1 is a kind of nuclear protein with transcriptional activity induced by hypoxia [95]. When mild and moderate ischemic hypoxia occurs, HIF-1 is upregulated and then promotes angiogenesis in the ischemic region by directly upregulating the expression of VEGF [96–99]. However, HIF-1 also has an opposite effect that is bad for functional repair following stroke. Several experiments have shown that upregulated HIF-1 could aggravate apoptosis, autophagy, oxidative stress, and inflammation [98, 100]. Although brain-derived neurotrophic factor (BDNF) is a neurotrophic protein that is widely expressed in the central nervous system and it can promote angiogenesis and neurogenesis [101], increased BDNF can promote the inflammatory response by increasing neutrophil infiltration [102]. Besides, the notch signaling pathway directly receives signals from neighboring cells, the signals are transmitted to the nuclear as well as activate the expression of related transcription factors and then regulate angiogenesis [103]. Likewise, notch signaling can also regulate inflammation in activated microglia in cerebral ischemia [104, 105].

In summary, the crosstalk between inflammation and angiogenesis following stroke relates to the interaction of various cells, cytokines, and signaling pathways in the central nervous system.

### 4.2. The Effects of Exosomes in Crosstalk between Inflammation and Angiogenesis following Stroke

**Figure 2:**
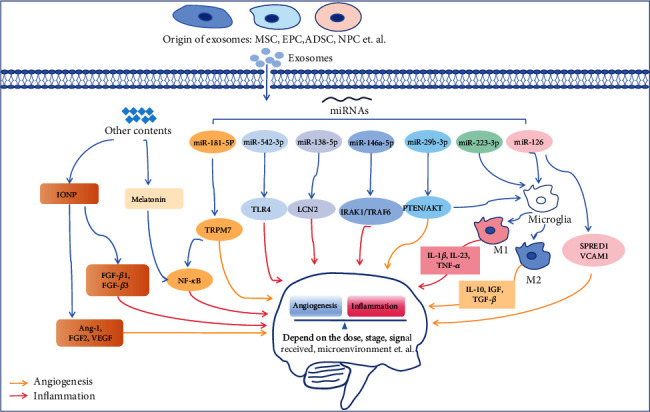
Summary of the exosomes in crosstalk between inflammation and angiogenesis. The red arrow represents regulation of inflammatory response, the yellow arrow represents regulation of angiogenesis, and the blue arrow represents general regulation. Exosomes from different sources carry miRNAs and other contents that affect central nervous system cells and simultaneously regulate inflammation and angiogenesis through different signals. The balance between inflammation and angiogenesis is also influenced by many factors following stroke, including the pathological stage of stroke, the signal received, and the dose of therapeutic substances. Abbreviations: MSC: mesenchymal stem cell; EPC: endothelial progenitor cell; ADSC: adipose-derived stem cell; NPC: neural progenitor cell; IONP: iron oxide nanoparticles; FGF: fibroblast growth factor; Ang-1: Angiogenin 1; VEGF: vascular endothelial growth factor; NF-*κ*B: nuclear factor-*κ*B; TRPM7: transient receptor potential cation channel member 7; TLR4: Toll-like receptor 4; LCN2: lipocalin 2; IRAK1: IL-1 receptor-associated kinases 1; TRAF6: TNF receptor-associated factor 6; PTEN: phosphatase and tensin homologue deleted on chromosome 10; AKT: protein kinase B; IL-1*β*: interleukin-1*β*; TNF-*α*: tumor necrosis factor *α*; TGF-*β*: transforming growth factor *β*; IGF: insulin-like growth factor (IGF); SPRED1: sprouty-related EVH1 domain-containing protein 1; VCAM1: vascular cell adhesion molecule 1

The circulatory system and immune systems have complex interactions, so some new therapeutic measures are needed to address the challenges of inflammation and angiogenesis in distinct environments. Exosomes can be used as a potential tool, which reprograms recipient cells by providing numerous factors of proangiogenesis and regulating inflammation such as nuclear acids, noncoding RNAs, and proteins. The effects of exosomes on crosstalk between inflammation and angiogenesis are shown in Figure 2.

#### 4.2.1. MSC-Derived Exosomes

The transplanted MSCs increase the vascular density in infarcted areas via upregulating the expression of angiogenic factors such as HIF-1, VEGF, and Angiogenin 1 (Ang-1). The improvement effects not only include the promotion of the maturation and stability of blood vessels, the activation of endothelial cells, vascular smooth muscle cells, and pericytes but also include the reduction of inflammation and vascular leakage [106–110]. Moreover, MSCs can attenuate the circulating immune cells including B-cells, natural killer cells, and T-cells, which provide an appropriate external environment for brain remodeling [111]. Similarly, MSC-derived exosomes were certificated that participate in angiogenesis and inflammation. For example, MSC-derived exosomes with miR-29b-3p promote angiogenesis via targeting phosphatase and tensin homologue deleted on chromosome 10 (PTEN)/AKT pathway [112], and the PTEN/AKT indicated an increased ratio of M2 polarization to M1 polarization and thus inhibiting inflammation [113]. MSC-derived and loaded cholesterol-modified miR-210 exosomes target the ischemic brain and promote angiogenesis by upregulating the integrin *β*3, VEGF, and CD34 [114]. The MSC-derived exosomes decorated with iron oxide nanoparticles (IONP) exhibit a significant therapeutic effect on ischemic stroke, this effect is connected with angiogenesis, anti-inflammation, and antiapoptosis [23]. Notably, the IONP-decorated exosomes not merely obtain a better targeting ability but also activate the phosphorylation of c-Jun N-terminal kinase (JNK) and upregulate the expressions of angiogenic factors (Ang-1, FGF2, and VEGF) and anti-inflammation factors (TGF-*β*1 and TGF-*β*3) [23]. Ischemic stroke may cause more severe dysfunction in aged humans; in an animal research, MSC-derived EVs reduced brain macrophage infiltrates in aged MCAO rats and the accumulation of microglia in young rats. Meanwhile, the EVs increased angiogenesis in young and aged rats [115]. A hypoxic environment can stimulate angiogenesis; similarly, hypoxic-treated MSC-derived EVs were indicated that effectively increase BMEC proliferation, migration, and tube formation and promote the postischemic survival of BMECs [116]. In addition, MSC-derived exosomes significantly increase the number of newly formed doublecortin (a marker of neuroblasts) and von Willebrand factor (a marker of endothelial cells) and enhance neurovascular remodeling following stroke [117].

#### 4.2.2. EPC-Derived Exosomes

The EPCs not only merely migrate to the injury zone, participate in the angiogenesis, and restore the integrity of BBB but also have an important role in regulating the inflammatory response, lessening the motor and neurological impairments associated with stroke pathology [19, 118]. EPCs promote endothelial regeneration by stimulating the proliferation and migration of endogenic endothelial cells via paracrine mechanisms, instead of direct differentiation into mature endothelial cells [119, 120]. As a key component of paracrine secretion, the EPC-derived exosomes have been confirmed to promote angiogenesis and improve the recovery of function [121]. In recent studies, Wang et al. observed that EPC-derived exosomes decrease infarct size and increase cerebral blood flow and cerebral microvascular density, especially the exosomes with enrichment miR-126 enhance the therapeutic efficacy [122], and miR-126 has been found that promote angiogenesis and inhibit inflammation by repressing sprouty-related EVH1 domain-containing protein 1 (SPRED1) and vascular cell adhesion molecule 1 (VCAM1) [123]. In addition, EPC-derived exosomes enriched with miR-137 could be against apoptosis and mitochondrial dysfunction in SH-SY5Y cells, and the protective efficacy might be connected with the cyclooxygenase 2 (COX2)/prostaglandin E2 (PGE2) signaling pathway [124], COX2 is expressed in inflammation and other pathological processes, and the beneficial effect of anti-inflammation is connected with inhibition of COX2.

#### 4.2.3. Adipose-Derived Stem Cell- (ADSC-) Derived Exosomes

The ADSCs are self-renewing pluripotent stem cells derived from adipose tissue, and ADSCs have the characteristics of easy selection, nonimmunogenicity, and low risk in teratogenesis and tumorigenesis. ADSCs also play an important role in angiogenesis and immune regulation [125]. It has been demonstrated that ADSC-derived exosomes with miR-181b-5p promoted the mobility and angiogenesis of BMECs by targeting the transient receptor potential cation channel member 7 (TRPM7) following oxygen-glucose deprivation [126], and TRPM7 is a major controller that produces proinflammatory cytokine and activates NF-*κ*B by transmitting Ca^2+^; notably, angiogenesis and vascular remodeling also depend on the Ca^2+^ signal [126–128]. Moreover, the ADSC-derived exosomes enriched miR-126 against cerebral ischemia injury by promoting angiogenesis after stroke; meanwhile, the exosomes inhibit microglial activation and inflammatory response induced by stroke [129].

To sum up, exosomes are a cell-based therapy and the biological functions of exosomes are closely related to the biogenesis, cargoes, and target cells. Current researches focus on the stem cell-derived exosome, whereas there are numerous immune cells such as microglia-derived exosomes for crosstalk between inflammation and angiogenesis following stroke which needs further investigation. The roles of exosomes in inflammation and angiogenesis following stroke are shown in Table 1.

## 5. Conclusions and Perspectives

Given the above, inflammatory cells and cytokines may promote angiogenesis at different stages after stroke, and angiogenic factors also may induce inflammation depending on many complicating factors such as the dose, signal received, stage of stroke, and microenvironment. The interaction between neuroinflammation and angiogenesis dictates the injury and repair processes following stroke. It is crucial to develop therapeutic measures that target multiple systems to maximize therapeutic efficacy. Numerous evidences have indicated that exosomes exert protective and restorative through regulating the interaction of angiogenesis and inflammation effects in stroke.

Although exosomes have shown great therapeutic potential in the treatment of stroke, many challenges need to be clarified before exosomes are used as a new method for stroke. ① How to prepare more exosomes? The secretion of exosomes is finite, which limits the clinical application. Therefore, scale production is essential. At present, the measures to obtain a large number of exosomes mainly include the addition of elicitors or drugs, change the culture conditions, and extrusion. However, these methods may change the exosomal contents and affect the therapeutic effects. ② How to increase the targeting of exosomes? The drawback of undecorated exosomes is the poor targeting, thereby yielding a poor therapeutic outcome. Engineering exosomes have better targeting; for example, the MSC-derived exosomes modified with IONP can drastically improve the ischemic-lesion targeting [23, 25]. But the preparation of engineered exosomes involves multiple reaction steps that may affect the exosomal surface structures and molecules, resulting in adverse reactions. ③ How do loading the required drugs and bioactive substances into exosomes? The existing methods have coincubation of parental cells with drugs, directly loaded into exosomes, and directly incubated with exosomes. Using the method of coincubation of parental cells with drugs to secrete engineered exosomes is simple to operate but can not control the efficiency of drug delivery. It is a widely used method that the drugs are loaded directly into exosomes such as electroporation. The process is the drug diffuse into exosomes following the formation of the temporary pores on the exosomal membrane by electric stimulation. However, the process may damage the membrane structure of exosomes and lead to drug leakage. In addition, the drugs directly incubated with exosomes can not affect the integrity of membrane structure, and the loading efficiency is very limited and requires a large dose of drugs.

As described above, the challenges, activity, and therapeutic effects of exosomes can be affected easily. Therefore, comprehensive consideration of the secretion, preparation and uptake of exosomes, in-depth understanding of molecular transfer mechanisms and biological characteristics, and selection of appropriate modification and loading methods are of irreplaceable significance to better play the role of exosomes.

## Figures and Tables

**Table 1:** The roles of exosomes in inflammation and angiogenesis following stroke.

Source	Cargoes	Recipient cell	Target molecules/pathways	Function	Ref.
MSCs	miR-542-3p	HA1800 cells (human glial cells)	TLR4	Prevent inflammatory response	[63]
MSCs	/	Astrocytes	Nrf2/NF-*κ*B	Reduce the reactive astrogliosis and inflammation	[64]
BMSCs	miR-138-5p	Astrocytes	LCN2	Inhibit inflammation	[65]
Plasma	miRNAs	/	TLR4/NF-*κ*B	Against inflammation and inflammasome-mediated pyroptosis	[66]
NPCs	miRNAs	BV2 microglia	MAPK	Inhibit inflammation	[67]
MSCs	miR-223-3p	BV2 microglia	Microglial M1 polarization	Inhibit inflammation	[74]
hUMSCs	miR-146a-5p	Microglia	IRAK1/TRAF6	Attenuate neuroinflammation	[75]
Serum	miR-27-3p	Microglia	PPAR*γ*	Aggravate cerebral inflammation and promote microglia activation	[76]
BMSCs	miR-221-3p	Neurons	ATF3	Attenuate neuroinflammation and apoptosis	[78]
MSCs	miR-29b-3p	BMECs, neurons	PTEN, AKT	Promote angiogenesis and antiapoptosis	[112]
MSCs	miR-210	Cerebral vascular endothelial cells	integrin *β*3,VEGF, CD34	Promote angiogenesis	[114]
MSCs	/	Pheochromocytoma 12 cells, human umbilical vein endothelial cells	Neuronal nitric oxide synthases, arginase-1, microtubule-associated protein 2, TNF-*α*, IL-1*β*, COX2	Enhance angiogenesis and anti-inflammatory	[23]
MSCs	/	/	/	Promote angiogenesis, reduce macrophage infiltrate and microglia accumulate	[115]
MSCs	miRNAs	BMECs	VEGF, leukocyte transendothelial migration	Induce angiogenesis	[116]
MSCs	/	Endothelial cells, neuroblasts	Doublecortin, von Willebrand factor	Increase angiogenesis and enhance neurovascular remodeling	[117]
EPCs	miR-126	BMECs, neurons, astrocytes, microglia	VEGFR2, caspase-3	Promote angiogenesis	[122]
EPCs	miR-137	SH-SY5Y cells	COX2/PGE2	Against apoptosis and mitochondrial dysfunction	
ADSCs	miR-181b-5p	BMECs	TRPM7	Promote angiogenesis	[126]
ADSCs	miR-126	Microglia, endothelial cells	TNF-*α*,IL-1*β*,von Willebrand factor	Promote angiogenesis, inhibit microglial activation and inflammatory response	[129]
